# The Good Treatment, the Bad Virus, and the Ugly Inflammation: Pathophysiology of Kidney Involvement During COVID-19

**DOI:** 10.3389/fphys.2021.613019

**Published:** 2021-03-11

**Authors:** Marie-Bénédicte Le Stang, Jordan Desenclos, Martin Flamant, Benjamin G. Chousterman, Nahid Tabibzadeh

**Affiliations:** ^1^Université de Paris, U1149 INSERM, Paris, France; ^2^Nephrology, Dialysis and Transplantation Department, CHU Clermont Ferrand, University Clermont Auvergne, Clermont Ferrand, France; ^3^Department of Physiology, Hôpital Bichat, FHU APOLLO, DMU Dream, APHP.Nord, Paris, France; ^4^INSERM U942 MASCOT, Université de Paris, Paris, France; ^5^Department of Anesthesia and Critical Care, Hôpital Lariboisière, FHU PROMICE, DMU Parabol, APHP.Nord, Paris, France

**Keywords:** COVID, viral sepsis, inflammation, AKI (acute kidney injury), kidney, SARS – CoV – 2, COVID–19

## Abstract

Kidney involvement is a common complication during SARS-CoV-2 infection. Its association with poor outcomes, especially in critically ill patients, raises issues whether kidney involvement reflects multi-organ damage or if it is a specific feature of the infection. Based on observational studies, autopsy series, and on current understanding of the route of entry of the virus, this review will highlight the different types of kidney involvement during COVID-19 and put them in the perspective of the different pathophysiological hypotheses. Virus entry route through ACE2 ligation and TMPRSS2 coligation allows identifying potential viral targets in the kidney, including tubules, endothelial cells, and glomerulus. While reports have described damages of all these structures and virus kidney tropism has been identified in renal extracts in autopsy series, no direct viral infection has been found in the latter structures thus far on kidney biopsies. Notwithstanding the technical challenge of disclosing viral invasion within tissues and cells, viral direct cytopathogenic effect generally does not appear as the cause of the observed renal damage. Inflammation and altered hemodynamics, described as “viral sepsis,” might rather be responsible for organ dysfunction, including kidneys. We shall place these various mechanisms into an integrated vision where the synergy between direct viral pathogenicity and systemic inflammation enhances renal damage. As SARS-CoV-2 inexorably continues its rampant spread, understanding the sequence of events in the kidneys might thus help inform improved therapeutic strategies, including antiviral drugs and immunomodulators.

## Introduction

Since the COVID-19 outbreak in January 2020, SARS-CoV-2 infection has affected so far more than 80 million people around the world with nearly 2 million reported deaths according to the WHO (World Health Organization [WHO], 2021). Although the most populous countries have gone through their first wave, the pandemic is still ongoing worldwide with some countries, including France, worryingly experiencing a recent outbreak in new cases and new admissions in hospital, notably in intensive care units (ICUs). Finding therapeutic strategies is thus of major importance to mitigate the impact of the pandemics, as measures of social distancing seem insufficient to contain the spread of the virus.

The main organ involvement during COVID-19 infection is the lung, but extrapulmonary manifestations are emerging not only as more frequent than initially hypothesized but also as of major impact during the clinical course of the infection. In particular, kidney impairment has been extensively reported and is associated with poor outcomes (Robbins-Juarez et al., 2020). However, pathophysiology of kidney involvement during SARS-CoV-2 infection remains to be elucidated. Indeed, mechanistic and experimental studies are still lacking and most of the hypotheses rely on observational retrospective clinical data, with frequently missing information, and some pathological findings that might give insights on potential mechanisms.

Kidneys are richly vascularized organs, as renal blood flow (RBF) accounts for 25% of cardiac output. While this high RBF allows efficient homeostasis of electrolytes and acid–base balance, the inevitable backlash is a high susceptibility to hemodynamic changes and systemic diseases, infectious or immune related (Maher, 1981). Even though the regulating system of tubuloglomerular feedback allows preserving glomerular filtration rate (GFR) in physiology, this refined adapting system cannot suffice in major pathophysiological states, such as that encountered during severe COVID-19.

Efficient as kidneys may be, they are consequently prone to be the target of various diseases and, as such, reflect these diseases and their severity. This paradigm should thus apply to SARS-CoV-2–related acute kidney injury (AKI), which reflects the systemic phase of the infection. However, whether the severity of the disease is due to viral dissemination and/or systemic inflammation is still a matter of debate.

Consequently, understanding kidney involvement might be the bridge to a better understanding of the disease itself. This might thus lead to optimal therapeutic targets, including antiviral and/or anti-inflammatory drugs.

In this view, we will focus on emerging data regarding kidney involvement during COVID-19 and infer pathophysiological hypotheses that might finally shed light on potential therapeutic interventions.

## Acute Kidney Injury and COVID-19

### Epidemiology

Acute kidney injury (AKI), defined by a rapid increase in serum creatinine and/or a sudden decrease in urinary output (KDIGO Guidelines, 2012), has been reported in several studies on COVID-19. The prevalence vary widely depending not only on the severity of the disease but also on geographical factors, and consequently the population studied (Robbins-Juarez et al., 2020): from 0.5% in the report from Guan et al. (2020) gathering findings from 1,099 outpatients and hospitalized patients throughout China, to 80% of patients while in ICU in the study from Rubin et al. (2020). Consistently, severe AKI requiring renal replacement therapy (RRT) also occurs more frequently in critically ill patients, in 40 to 55% of cases (Chand et al., 2020; Mohamed et al., 2020) compared with a general prevalence of 0.8 to 14.7% in the meta-analysis gathering 20 reported cohorts from Robbins-Juarez et al. (2020).

Acute kidney injury also seems to be close-related to the temporal evolution of pulmonary signs (Hirsch et al., 2020). Specifically, AKI and mechanical ventilation seem intricately linked in several reports. For example, Hirsch et al. (2020) reported that AKI occurred in 89.7% of patients on mechanical ventilation, compared with only 21.7% of the non-ventilated patients, and that almost all (96.8%) the patients of their cohort requiring RRT were on ventilator support. These results suffer, however, from an immortality bias as patients on mechanical ventilation must survive enough to be integrated in the analysis (Jamme and Geri, 2020). Besides mechanical ventilation, other commonly identified risk factors for AKI are age, male sex, and pre-existing comorbidities [cardiovascular disease, diabetes mellitus, hypertension, and chronic kidney disease (CKD)] (Fu et al., 2020), as well as black race and obesity (Bowe et al., 2020). In several studies, kidney involvement has been reported as an independent risk factor of mortality, with a meta-analysis from Fu et al. (2020) showing a pooled risk ratio of mortality from 142 studies of 4.6 (95% CI 3.3–6.5) compared with patients with no AKI. Other events undeniably contribute both to general deterioration and to AKI and are not taken into account in these analyses. We thus cannot exclude residual confounding factors such as hemodynamic instability during the course of the disease, degree of hypoxemia, or septic events.

### Features of Kidney Involvement During SARS-CoV-2 Infection: Are They Specific to COVID-19?

Kidney involvement in COVID-19 usually presents with non-specific features of AKI: rise in serum creatinine and/or decrease in urine output. The presence of AKI does not prejudge the cause of kidney damage and might exist regardless of the underlying etiology. Authors have reported general features of kidney impairment during COVID-19, as well as more specific descriptions and histopathological data in case series or case reports. However, the precise rate of specific tubular, glomerular, and vascular involvement is still unknown, and whether they represent key features of SARS-CoV-2–related kidney injury remains to be determined.

#### Prerenal Azotemia

The effective decrease of extracellular volume, induced by poor intake of water and food, high fever, diarrhea, and ultimately hypovolemic, cardiogenic, or septic shock observed in the course of COVID-19, might induce a decrease in renal blood flow resulting in GFR decrease. Moreover, rapid recovering of AKI after volume supplementation has been described in 12.2% of critically ill patients (Xia et al., 2020), which might conduct retrospectively to the diagnosis of prerenal acute kidney failure. When measured, a fractional excretion of urinary sodium <1% was observed in 38% of cases upon admission in ICU (Mohamed et al., 2020).

It is interesting to note that among patients with severe COVID-19 admitted to ICUs, AKI is frequently associated with invasive ventilation (Hirsch et al., 2020). As a matter of fact, patients with invasive ventilation display altered abdominal pressure, especially when they are exposed to high expiratory pressure during mechanical ventilation. In this setting, the mechanical ventilation–induced high abdominal pressure compromises abdominal venous drainage, resulting in renal venous congestion, which is a prominent cause of ischemic kidney injury. Mechanical ventilation is also correlated with more profound and longer hypoxemia, the latter potentially resulting in sustained renal ischemia. Other factors have been suggested to contribute to kidney injury during invasive ventilation, including neurohormonal changes and inflammatory mediators (Koyner and Murray, 2008).

#### Acute Tubular Injury: Is Tubular Injury A Specific Feature of COVID-19 and Does it Reflect Viral Invasion?

Kidney involvement during COVID-19 usually presents with features of tubular injury: mild proteinuria, in 15.5 (Xia et al., 2020) to 69% of patients (Cheng et al., 2020; Mohamed et al., 2020; Pei et al., 2020), of low molecular weight when assessed (Werion et al., 2020), tubular casts, and renal tubular epithelial cell casts on urine sediment microscopic analysis (Hernandez-Arroyo et al., 2020). Based on these findings and others, several authors suggest that a direct and specific tubular viral invasion occurs during SARS-CoV-2 infection. There are some limitations in this interpretation that we will summarize in this section.

##### Is There a Specific Fanconi Syndrome During SARS-CoV-2-Related AKI?

Fanconi syndrome is a specific proximal tubular dysfunction characterized by abnormal handling of solutes that are secreted and/or reabsorbed by proximal tubule. Fanconi syndrome features are the following:

##### Tubular proteinuria

Low molecular weight proteinuria and/or urinary albumin/protein ratio <50% are key features of acute tubular injury, irrespective of the cause of tubular damage. Consistently, tubular injury markers such as NGAL and KIM-1 have been extensively studied to better detect acute tubular injury before the increase in serum creatinine and the decrease in estimated GFR (Parikh et al., 2010). Consequently, low molecular weight proteinuria cannot be considered a specific feature of Fanconi syndrome.

##### Aminoaciduria, uric acid and phosphate renal wasting, normoglycemic glycosuria

Two reports have studied these parameters in COVID-19 patients. Werion et al. (2020) found unquantified aminoaciduria in 6 out of 13 tested SARS-CoV-2–infected patients, 18/39 with hypouricemia and fractional excretion of uric acid (FeUA) > 10%, and 6/32 with hypophosphatemia and fractional excretion of phosphate >20%. Kormann et al. (2020) found hypouricemia and FeUA > 10% in 14/35 patients, a calculated maximal threshold for phosphate reabsorption (TmPi/GFR) <0.77 mmol/L in 19/48 patients, and, conversely to Werion et al., normoglycemic glycosuria in 11/28 patients.

##### Specific cautions in proximal tubular functions interpretation during AKI

First, in the same line with tubular proteinuria, proximal tubular transports, especially sodium co-transporters (including sodium-phosphate co-transporters), are disturbed during AKI in experimental models as well as in humans (Basile et al., 2012; Vallon, 2016). Second, FeUA might be increased in patients with volume overload as frequently seen in critically ill patients (Kazory et al., 2020); this situation has even been described during the first SARS outbreak, where it was associated with inflammatory cytokines (Wu et al., 2005). Third, phosphatemia is often decreased in critically ill patients, mostly due to an intracellular transfer mechanism, falsely decreasing the TmPi/GFR calculation while phosphate tubular transport is not affected (Suzuki et al., 2013). Finally, proximal tubules are the mainstay of injury during AKI (Takaori et al., 2016); hence, it should not be surprising to find these features during any type of acute tubular injury. However, as these markers are not usually assessed in acute tubular injury, it remains uncertain whether they are more frequent during SARS-CoV-2–related AKI.

##### Specific cautions in urinary biochemistry interpretation during AKI

Another limitation of these findings is the possible flaws in urine biochemistry during acute illness. First, during AKI, urine creatinine concentration rapidly decreases. Ratios based on its level might thus be inaccurate. In the same line, fractional excretion of all the solutes will appear elevated even with no modification of tubular handling. Finally, proteinuria can increase in conditions such as fever, oliguria, or hematuria in patients with urine catheter (Nguyen et al., 2009; Gabarre et al., 2020).

#### Could SARS-CoV-2–Related AKI Be a Toxic Acute Tubular Injury?

##### Rhabdomyolysis

Some authors have reported results suggestive of a contribution of myoglobin to kidney damage. Mohamed et al. (2020) found high levels of plasmatic creatine phosphokinase (CPK), above 1000 U/L in more than 30% of their patients. In most cases, values were not as high as in typical rhabdomyolysis-induced AKI (above 15,000 IU/ml) (Bosch et al., 2009). Pigmented casts were also found in 3 out of 26 autopsy analysis of kidney tubules from deceased patients with high CPK levels (Su et al., 2020). The association of myoglobinuria with dehydration, sepsis, and acidosis might thus trigger AKI in a subpopulation of SARS-CoV-2–infected patients.

##### Toxic acute tubular injury

A high incidence of AKI, especially in critically ill patients, along with a lack of efficacy on primary endpoints during preliminary data analysis has led the Discovery trial investigators to an early termination of inclusions in the lopinavir/ritonavir arm (Inserm, 2020). While it was not confirmed by the lopinavir/ritonavir versus standard of care randomized trial by Cao et al. (2020), Binois et al. (2020) found an association between AKI and this treatment regimen in patients admitted in their ICU unit, notwithstanding potential confounding factors, as this study involved critically ill patients with features of viral sepsis. Interestingly, Arrestier et al. (2020) explored three critically ill patients with AKI while on lopinavir/ritonavir treatment and found neither urinary crystals nor evidence of drugs by infrared spectroscopy analysis of urinary sediment. Conversely, they mostly found cellular debris and granular casts. Overall, these results suggest that during SARS-CoV-2 infection, potentially nephrotoxic treatments might contribute to AKI on underlying subclinically damaged kidneys.

#### SARS-CoV-2–Related Acute Tubular Injury: Other Pathophysiological Hypotheses

##### Ischemic ATI

Prerenal azotemia often overlaps with ischemic tubular injury or might rapidly evolve toward organic tubular damage when hemodynamic changes are severe. Several other factors, in particular systemic inflammation, microvascular damage, and reduction in kidney medullary perfusion, contribute to parenchymal injury during ischemic ATI (Basile et al., 2012).

##### Viral renal invasion

As far as we currently know, establishing that SARS-CoV-2–related AKI is related to viral invasion needs simultaneous ACE2 (type 2 angiotensin-converting enzyme) and TMPRSS2 (transmembrane protease, serine 2) expressions in the same site, and detection of viral RNA in those tissues during infection. Indeed, host cell entry of SARS-CoV-2 involves two major steps: binding of the Spike (S) protein to ACE2 and cleavage in two subunits (S1 and S2) by the host TMPRSS2, thus initiating fusion and endocytosis of the virus (Batlle et al., 2020). In both rodent and human kidneys, ACE2 protein and transcript are highly expressed in the proximal tubule, in parietal and visceral epithelial cells of the glomerulus, in vascular smooth muscle cells, and in the endothelium of interlobular arteries (Lely et al., 2004; Ye et al., 2006; Batlle et al., 2020). TMPRSS2 is expressed at lower levels in the proximal tubule and the glomerulus compared with distal nephron, questioning the kidney infectivity of SARS-CoV-2, and raising the possibility of SARS-CoV-2 priming by other TMPRSS subtypes. Of note, Pan et al. (2020) have found differential expressions of ACE2 and TMPRSS2 in Asian and European populations, potentially explaining different susceptibility to SARS-CoV-2–related AKI.

Viral RNA and proteins have been extensively reported in upper respiratory tract and pulmonary cells, by various direct techniques including spatial identification (Best Rocha et al., 2020; Bussani et al., 2020; Ehre, 2020; Hou et al., 2020; Schaefer et al., 2020). In contrast, findings are far more conflicting in the kidneys. Viral RNA has been found in 40 to 78% of studied kidney extracts in autopsy series (Bradley et al., 2020; Braun et al., 2020; Edler et al., 2020; Puelles et al., 2020; Remmelink et al., 2020; Wichmann et al., 2020), with similar viral loads in the liver and the heart, which appear to be substantially lower than in respiratory samples. It correlates with viremia when assessed (Wichmann et al., 2020), but not always with clinical and histopathological findings. Kidney histology of these autopsy series found either normal tissue or aspecific shock lesions, tubular injury, and autolysis (Bradley et al., 2020; Edler et al., 2020; Remmelink et al., 2020; Santoriello et al., 2020; Wichmann et al., 2020). Interestingly, a high proportion of chronic vascular and glomerular lesions has also been reported. Braun et al. (2020) reported the presence of pre-mortem AKI in 23 out of 32 patients with SARS-CoV-2–positive kidney samples. A report in May 2020 by Puelles et al. (2020) suggested the presence of SARS-CoV-2 RNA and protein, respectively, by *in situ* hybridization (ISH) and immunofluorescent staining within podocytes, glomerular endothelial cells, and tubular cells, whereas none of the more recent kidney biopsy series found evidence of viral RNA with validated techniques including ISH (Couturier et al., 2020; Kudose et al., 2020; Sharma et al., 2020). Consistently, the main finding in these kidney biopsy series is acute tubular injury. Besides these conflicting results, the presence of virus in cells and tissues does not imply that there is a cytopathogenic infection, as demonstrated by *in vitro* studies by Eckerle et al. (2013) showing the absence of SARS-CoV infectivity in kidney epithelial cells.

###### Specific cautions in interpreting indirect ultrastructural evidence of virus

Upon electron microscopic analysis of kidney structures, some authors have interpreted the presence of intracellular inclusions as direct evidence of the presence of SARS-CoV-2. Since then, several authors have disclosed these non-specific microvesicular bodies in biopsies from non-infected patients with various disorders (Calomeni et al., 2020; Cassol et al., 2020; Goldsmith and Miller, 2020). Consequently, intracellular inclusions should not be considered as viral inclusions if not associated with specific virus identification techniques.

##### Viral septic AKI

On the whole, even though viral RNA is found in kidneys of autopsies, current evidence does not support a major role of direct viral pathogenicity on the kidneys. Yet, severe viral infections, in particular with respiratory viruses, can induce multi-organ damage, including acute respiratory distress syndrome (ARDS) and AKI (Gu et al., 2020). Before the recent outbreak of COVID-19, public health concerns about mortality during influenza viruses, SARS-CoV, and MERS-CoV infections have yielded increasing interest in on “viral sepsis,” defined as a virus-related “life-threatening organ dysfunction resulting from dysregulated host responses to infection” (Singer et al., 2016). This implies that organ damage does not directly depend on viral invasion and SARS-CoV-2–related local inflammation, but is rather considered as a remote inflammation, due to pulmonary involvement resulting in a massive systemic response that is deleterious in itself. This crosstalk between distant organs is likely mediated by several factors including cytokine and DAMP (damage-associated molecular pattern) release by injured tissues. Consistently with this hypothesis, patients with severe COVID-19 often present with multi-organ and hemodynamic failure, which appears late in the time course of the infection. This presentation is often associated with a pro-inflammatory phenotype (Azoulay et al., 2020), including fever, high levels of C-reactive protein, and high levels of pro-inflammatory cytokines, particularly IL-6 (Chen et al., 2020), which is an established mediator of AKI in experimental models (Nechemia-Arbely et al., 2008). Besides, renal blood flow decrease has also been demonstrated in COVID-19 patients and was comparable with patients with bacterial sepsis in a case–control study (Watchorn et al., 2020). Consequently, sepsis-associated hemodynamic changes and inflammation might be of major importance in the pathophysiology of SARS-CoV-2–related AKI.

##### Specific cautions in extrapolating from pulmonary endothelial dysfunction and hypercoagulability to the kidney injury

Pulmonary hypercoagulability is indeed a major feature during COVID-19, raising the issue of endothelial dysfunction (Ackermann et al., 2020) as part of the viral sepsis, or as a distinct and specific mechanism of multi-organ damage (Kaur et al., 2020; Pons et al., 2020). Indeed, hypercoagulability and endothelial dysfunction are interrelated especially in the setting of thrombo-inflammation (Abou-Ismail et al., 2020). However, endotheliitis and thrombosis have been mostly described in lungs, and systemic hypercoagulability is rather occasional based on published data (Klok et al., 2020). Regarding kidneys, two cases of multiple renal infarctions have been reported (Post et al., 2020), occurring in both cases simultaneously with general clinical worsening; renal outcome eventually happened to be favorable along with respiratory and general improvement.

Renal microvascular or endothelial involvement seems rare as to date few cases of thrombotic microangiopathy (TMA) have been reported to be unequivocally related to COVID-19 (Akilesh et al., 2020; Jhaveri et al., 2020; Sharma et al., 2020). In autopsy series, Santoriello et al. (2020) found focal fibrin thrombi in only 6 out of 42 autopsies, and Varga et al. (2020) found lymphocytic endotheliitis in the kidney of one out of three deceased patients, with no precision as to which renal vascular structure was involved. These results suggest that if present, endothelial lesions are mild and do not account for the majority of SARS-CoV-2–related AKI. In the same line, while complement activation has been suggested as a major contributor to endothelial dysfunction and hypercoagulability, there is currently no evidence of such mechanism in the kidney, as no specific complement mediated renal lesions have been reported so far (such as membranoproliferative glomerulonephritis or C3 glomerulopathy). In the aforementioned published case of TMA, however, a comprehensive complement testing showed a slightly decreased level of circulating factor H, and increased circulating CBb and SC5b-9 levels, suggesting an activation of the alternative pathway of the complement in this specific case, in which genetic testing was not performed (Jhaveri et al., 2020).

### The Particular Case of COVID-19–Associated Collapsing Glomerulopathy

Although probably rare, glomerular involvement seems a characteristic feature during SARS-CoV-2 infection (Table 1). It presents with severe AKI and heavy proteinuria, with a nephrotic syndrome (i.e., with hypoalbuminemia) in a majority of cases. Hematuria is inconstant. Histopathological findings are those of a collapsing glomerulopathy, which is a variant form of focal segmental glomerulosclerosis associated with poor renal prognosis. This lesion can be observed in other viral-associated nephropathies (HIV, CMV, EBV, and Parvovirus B19) (Velez et al., 2020). In particular, it has been described in HIV patients from African origin expressing risk variants for APOL1 gene. When tested, all the cases of collapsing glomerulopathy during COVID-19 occurred in patients with the same APOL1 risk variants, suggesting a common underlying susceptibility and a common second-hit mechanism (Wu et al., 2020). Noteworthy, none of the published cases found SARS-CoV-2 viral RNA in the injured glomeruli using validated direct identification techniques (PCR, ISH, and IHC). Conversely, Wu et al., found increased *in situ* chemokine gene expression consistent with electron microscopy findings of endothelial reticular aggregates often associated with conditions presenting with elevated α-interferon (Gaillard et al., 2020).

**TABLE 1:** Summary of the type of reported kidney involvement during SARS-CoV-2 infection according to the underlying site of kidney damage.

Renal involvement	Features	Underlying condition	References	Commentaries
**Prerenal azotemia**	AKI Signs of ECV decrease FeNa <1% RBF decrease Favorable outcome after volume repletion	***Hemodynamic changes*** Hypovolemia Venous congestion Mechanical ventilation	[Bibr B19][Bibr B74][Bibr B68][Bibr B46]	
**Tubular**	AKI Low-range proteinuria Low molecular weight proteinuria ±Hypouricemia ±Hypophosphatemia ±Aminoaciduria	***Ischemic ATI Sepsis-associated ATI*** *Rhabdomyolysis*	[Bibr B69][Bibr B41][Bibr B46][Bibr B43][Bibr B58][Bibr B60]	No direct identification of SARS-CoV-2 (ISH, IHC, and PCR) (Unspecific microvesicular bodies on electron microscopy)
**Glomerular**	AKI Nephrotic-range proteinuria Albuminuria ±Hematuria	***Collapsing glomerulopathy*** *Membranous nephropathy Minimal change disease Anti-GBM GN Pauci-immune crescentic GN* ***Chronic glomerulosclerosis***	[Bibr B43][Bibr B58][Bibr B60][Bibr B72][Bibr B28]	*APOL-1* variant–associated collapsing glomerulopathy Role of interferon?
**Vascular**	AKI Hematuria ±Low-range proteinuria Severe COVID-19	*Microvascular* 6 cases of TMA Focal fibrin thrombi in 6/42 *Macrovascular* 2 cases of renal infarction ***Chronic vascular lesions***	[Bibr B37][Bibr B4][Bibr B58][Bibr B53]	Evidence of complement activation in one case of TMA Evidence of multiple thrombosis in one case of renal infarction

*In bold the most frequently reported lesions. AKI, acute kidney injury; ECV, extracellular volume; ATI, acute tubular injury; ISH, *in situ* hybridization; IHC, immunohistochemistry; GBM, glomerular basal membrane; GN, glomerulonephritis; TMA, thrombotic microangiopathy.*

## Conclusion: Pathophysiologic Hypotheses and Therapeutic Perspectives

**FIGURE 1:**
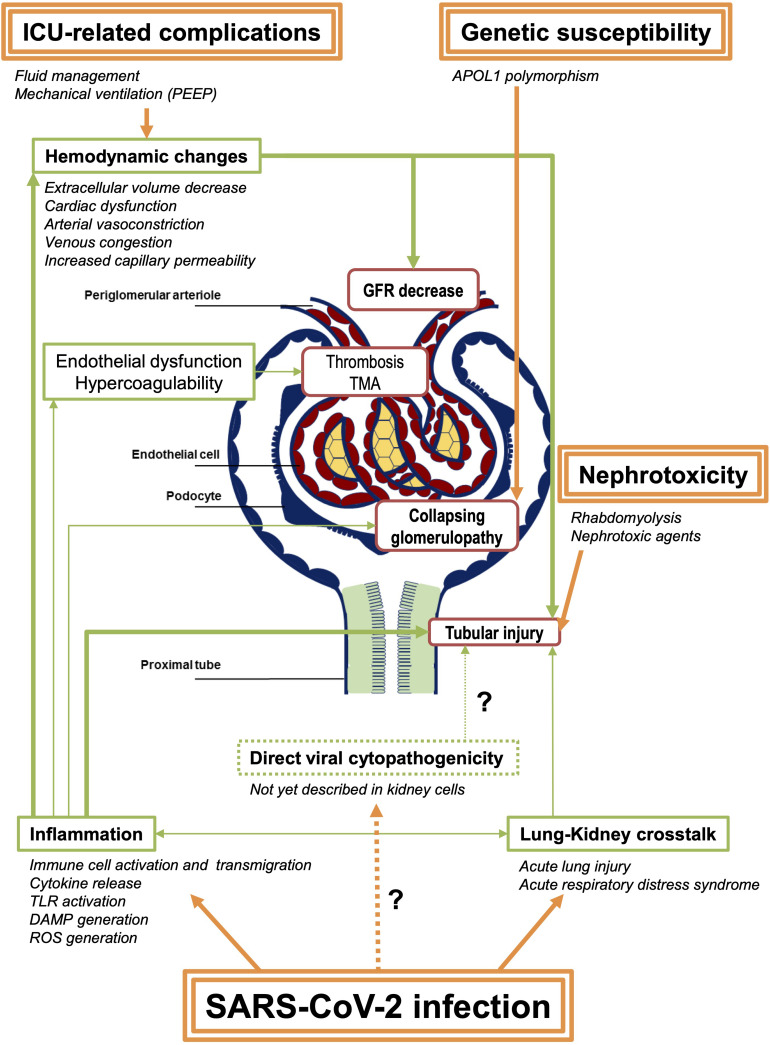
Summary of the potential pathophysiological mechanisms of SARS-CoV-2–associated AKI. SARS-CoV-2 infection induces direct pulmonary injury that might lead to systemic inflammation. Hemodynamic changes are also frequent in patients admitted in ICU, due to the infection and its complication, as well as to medical interventions. These modifications result in GFR decrease and thus prerenal azotemia, potentially leading to acute ischemic tubular injury. Other factors including inflammation itself and tubular toxicity due to nephrotoxic agents (antibiotics, antiviral drugs, etc.) contribute to acute tubular injury in these patients. Few cases of TMA and renal vascular thrombosis have also been reported, raising the hypothesis of endothelial dysfunction and systemic hypercoagulability in the most severe patients. Collapsing glomerulopathy is a specific feature of SARS-CoV-2–related AKI, also called COVAN (COVID-associated nephropathy) in reference to HIVAN (HIV-associated nephropathy), as they probably share common mechanisms, including the strong association with APOL1 genetic variants. Finally, following the report of the autopsy series from Puelles et al. (2020), a direct tubular or glomerular viral invasion has not yet been confirmed in other reports. Consequently, this mechanism remains controversial. Arrows in bold represent the proposed major mechanisms. PEEP, positive end-expiratory pressure; GFR, glomerular filtration rate; TMA, thrombotic microangiopathy; TLR, Toll-like receptors; DAMP, damage-associated molecular patterns; ROS, reactive oxygen species.

On the whole, current evidence does mostly not support the direct role of SARS-CoV-2 viral invasion in the pathophysiology of SARS-CoV-2–related AKI, even in the severe cases with systemic symptoms. Systemic complement activation is not corroborated either thus far. Rather, emerging knowledge of renal involvement during COVID-19 suggests that a state of viral sepsis results in acute tubular injury with concurrent hemodynamic changes and systemic inflammation, potentially aggravated by nephrotoxic treatments and myoglobinuria (Figure 1). Based on these published data on kidney involvement, in the setting of severe or late-stage SARS-CoV-2 infection, antiviral drugs and complement inhibitors might not be effective, at least if administered alone. Strict fluid management, eviction when possible of nephrotoxic agents, and hemodynamic control as well as anti-inflammatory or immunomodulatory drugs, contrariwise, might be promising in these situations.

Future experimental studies and interventional trials should unravel the natural history of SARS-CoV-2 infection and the best therapeutic options.

## Author Contributions

M-BL, NT, and BC wrote the outline of the review. M-BL and NT wrote the first draft of the manuscript and drafted the table. JD wrote the first draft of the table and edited the manuscript. MF contributed to the revised draft of the manuscript and new figure. BC edited the manuscript, table, and figure. All authors edited and approved the final version of the manuscript.

## Conflict of Interest

The authors declare that the research was conducted in the absence of any commercial or financial relationships that could be construed as a potential conflict of interest.
